# Potential Approaches *Versus* Approved or Developing Chronic Myeloid Leukemia Therapy

**DOI:** 10.3389/fonc.2021.801779

**Published:** 2021-12-15

**Authors:** Emanuela Andretta, Caterina Costa, Consiglia Longobardi, Sara Damiano, Antonio Giordano, Francesco Pagnini, Serena Montagnaro, Massimiliano Quintiliani, Chiara Lauritano, Roberto Ciarcia

**Affiliations:** ^1^ Department of Veterinary Medicine and Animal Productions, University of Naples “Federico II”, Naples, Italy; ^2^ Cell Biology and Biotherapy Unit, Istituto Nazionale Tumori-IRCCS-Fondazione G. Pascale, Naples, Italy; ^3^ Department of Mental, Physical Health and Preventive Medicine, University of Campania “Luigi Vanvitelli”, Largo Madonna delle Grazie, Naples, Italy; ^4^ Department of Medical Biotechnologies, University of Siena, Siena, Italy; ^5^ Sbarro Institute for Cancer Research and Molecular Medicine, Center of Biotechnology, College of Science and Technology, Temple University, Philadelphia, PA, United States; ^6^ Unit of Radiology, Department of Medicine and Surgery, University of Parma, Parma, Italy; ^7^ Department of Life, Health and Environmental Sciences, University of L’Aquila, Naples, Italy; ^8^ Marine Biotechnology Department, Stazione Zoologica Anton Dohrn, Naples, Italy

**Keywords:** chronic myeloid leukemia, tyrosine kinase inhibitors, quiescent leukemia stem cells, Non-BCR-ABL targeted drugs, oncolytic therapy, antioxidants, exosomes, marine organisms

## Abstract

Tyrosine kinase inhibitors (TKIs) have revolutionized the treatment of patients with chronic myeloid leukemia (CML). However, continued use of these inhibitors has contributed to the increase in clinical resistance and the persistence of resistant leukemic stem cells (LSCs). So, there is an urgent need to introduce additional targeted and selective therapies to eradicate quiescent LSCs, and to avoid the relapse and disease progression. Here, we focused on emerging BCR-ABL targeted and non-BCR-ABL targeted drugs employed in clinical trials and on alternative CML treatments, including antioxidants, oncolytic virus, engineered exosomes, and natural products obtained from marine organisms that could pave the way for new therapeutic approaches for CML patients.

## 1 Introduction

Chronic myeloid leukemia (CML) is caused by a t (9;22) (q34; q11) reciprocal translocation resulting in the fusion between the breakpoint cluster region (*BCR*) and Abelson leukemia (*ABL1*) genes (1). The resultant BCR-ABL fusion protein, is a hyper-activated tyrosine kinase. The target-therapy is mainly based on the use of tyrosine kinase inhibitors (TKIs) that can inhibit this fusion protein’s activity (1).

Currently, the annual incidence of CML varies from 0.7 to 1.0 cases per 100 000 inhabitants, it increases with age and is higher in men than women with a ratio ranging from 1.2- 1.7 (2). CML occurs in the chronic phase (CML-CP) associated with massive expansion of myeloid cells. Acquisition of genetic mutations in *BCR-ABL1* and/or epigenetic alterations results in progression of the disease to an advanced phase classified as accelerated (CML-AP) or blastic (CML-BP) (3). In most classification systems, the following criteria are considered for a differential diagnosis between AP and BP: the percentage of blasts or basophils, white blood cells and splenomegaly unresponsive to therapy (4).

The development of TKIs has been crucial role in increasing the survival of patients in *chronic-phase* and limiting the disease progression. However, some limitations of TKIs treatment are related to the accumulation of *BCR-ABL1* mutations, which leads to the initial and persistent resistance to TKIs dependent to BCR-ABL. Furthermore, TKI-therapy is not effective on quiescent leukemia stem cells (LSCs) as they exhibit independent BCR-ABL TKI resistance (5). It was demonstrated that there is a connection between the quiescent LSCs and the CML progression as their genetic instability favors the accumulation of mutations, stimulating the progression of the disease to *accelerated* and *blastic*-*phase* (6). The molecular pathways altered in CML, which include the JAK/STAT, NF-kB, WNT/β-catenin, PI3K/AKT/mTOR, Hedgehog and NOTCH pathways, are involved in LSCs maintenance and resistance to TKIs (7). In addition to the side effects of TKIs approach (8), this therapeutic strategy has the following disadvantages: the high costs of TKIs and healthcare, of molecular monitoring of patients during the therapy (9) and teratogenicity (10).

Several studies are focused on the achievement of the treatment-free remission (TFR), a condition aiming to the interruption of TKI therapy in CML patients without recurrence of the disease. The recent guidelines recommend TKI discontinuation in CML-CP patients pre-treated for at least 3 years who exhibited molecular response with BCR-ABL1 level of < 0.01% or a 4-log reduction in transcription level respect to the baseline (MR4) for at a minimum 2 years (11). The efficacy of TFR seems related both to the duration of treatment with TKIs and the persistent molecular response. During the first 6 months after TKIs discontinuation, a deeper monitoring of patients is required to identify molecular relapse (11). Conversely, it was observed that only 14% of the total molecular recurrences occurred in follow-up CML patients after 2 years from TKIs discontinuation (12). Although further investigations are required in order to better clarify the failure of TFR, the level of natural killer cells and the presence of factors that stimulate the resistance of LSCs could affect the time of TFR (13). However, it is necessary to restart with TKI-therapy when molecular recurrences are detectable in patients (11). Here, we discuss the mechanisms underlying TKIs-resistance and we provide an update on current and innovative therapeutic approaches as strategies for CML therapy, focusing on *in vitro* and *in vivo* findings.

## 2 Overview of Conventional TKI Treatment

**Figure 1:**
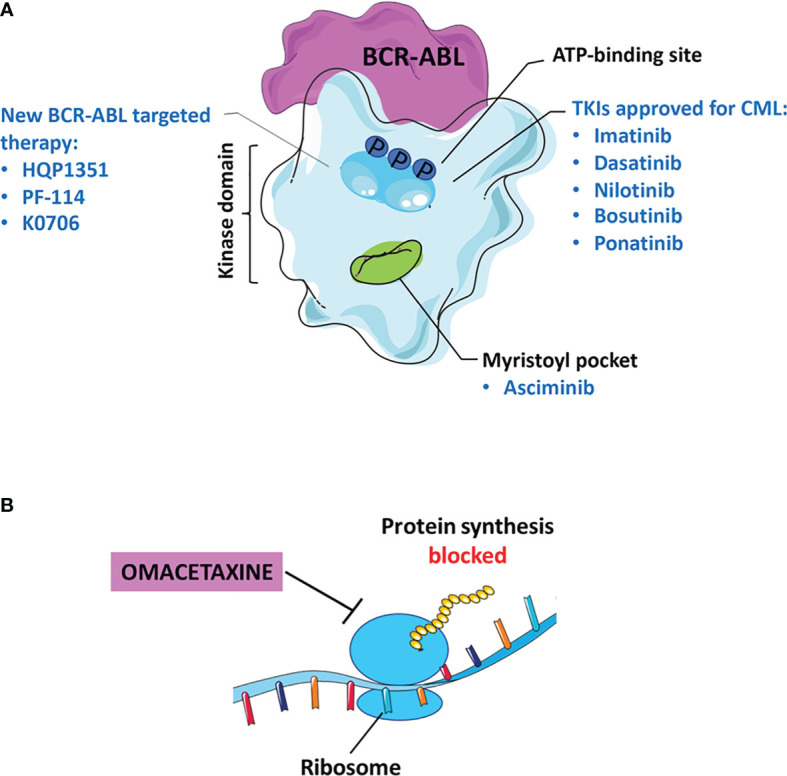
Current status of TKI therapy for CML. **(A)** Most approved drugs recognize the binding site for ATP in BCR-ABL, as: Imatinib, Dasatinib, Nilotinib, Bosutinib, and Ponatinib. Asciminib binds to the myristoyl pocket of the BCR-ABL kinase. The new molecules of *third* and *fourth generation* TKIs including: Olverembatinib (HQP1351), Vodobatinib (K0706) and PF-114. **(B)** Omacetaxine is an approved inhibitor that blocks protein synthesis.

Tyrosine kinase inhibitors (TKIs) have improved survival of patients with CML. Imatinib (*Gleevec*), a drug of first-line in the therapy of CML, was approved by *Food and Drug Administration* (FDA) in 2001 for accelerated phase and subsequently for newly diagnosed CML-CP patients. TKIs recognize the binding site for ATP in BCR-ABL kinase but with different affinity related to eventual mutations occured (**Figure 1A**). The missense and frameshift mutations in this site are related to Imatinib-resistance development (14, 15). For this reason, *second* and *third-generation* TKIs were developed: Dasatinib (*Sprycel*), Nilotinib (*Tasigna*), Bosutinib (*Bosulif*) and Ponatinib (*Iclusig)* (16, 17). In particular, *European Medicines Agency* (EMA) approved Dasatinib and Bosutinib in all three phases of CML while Nilotinib only during the chronic and accelerated phase (18–21 ^1^^–^^3^). In the **Table 1**, we report the known BCR-ABL mutations responsible for drug resistance in patients. By evaluating the efficacy of *second-generation* TKIs versus Imatinib in peripheral blood leukocytes derived from newly diagnosed patients, these drugs significantly induced apoptosis more than Imatinib (43). In addition, these drugs were tested in clinical trials for newly diagnosed CML-CP patients. After a follow-up of 12 months with Dasatinib (44), Nilotinib (45) or Bosutinib (46), significantly higher rates of major molecular response (MMR) and complete cytogenetic response (CCyR) have been observed (NCT00481247; NCT00471497; NCT02130557). The most common side effects are pleural effusion occurring after Dasatinib treatment (47, 48), headache and skin rashes more frequent with Nilotinib compared to Imatinib (45). Increased transaminase levels and diarrhea are observed in patients treated with Bosutinib respect to Imatinib (46). According to guidelines, the use of *second-generation* TKIs is recommended for patients without comorbidities over Imatinib monotherapy (8) in order to obtain a faster response and thus make the patients eligible for TFR (11). It is necessary to re-initiate the therapy when molecular relapse occurs (11). A new selective TKI is Radotinib (IY5511HCl), currently approved in Korea for CML-CP patients with newly diagnosis or not responsive to other TKIs (49). The efficacy and safety of Radotinib were demonstrated in a phase II study (NCT01602952). After treatment with this TKI, 65% of enrolled patients had a major cytogenetic response (MCyR) and a CCyR in 47% of them. The most common hematological side effects are thrombocytopenia (24.7%) and anemia (5.2%); while the non-hematological ones are fatigue (3.9%), asthenia (3.9%) and nausea (2.6%) (50). Compared to Imatinib, Radotinib showed superior efficacy with 91% CCyR versus 77% and induced MMR in 52% of patients (NCT01511289) (51).

**Table 1:** The main TKIs approved for CML patients during the various phases of the disease, their molecular targets and BCR-ABL mutations responsible for drug resistance in CML patients.

Drugs	Molecular target of the drugs	Indications of drug use for CML patients at various phases of the disease	BCR-ABL mutations responsible for drug resistance in patients	Ref
Imatinib	BCR-ABL	First line TKI in CML (all phase)	Y253F/H; E255K; M351T; F359V; T315I; F317L; Q252H/R	(22–26^1^)
		For adult and children with a new diagnosis of CML or not eligible for bone marrow transplant.		
Nilotinib	BCR-ABL	For CML-CP and -AP patients resistant or intolerant to other options, such as Imatinib.	T315I	(27–30^2^)
		For newly diagnosed CML-CP patients.		
Dasatinib	BCR-ABL/Src	First line TKI for newly diagnosed CML patients.	Less sensitive mutations: Y253H; E255V/K, F359V/CT315I	(28, 31–33)
		For resistance or intolerance to other drugs, including Imatinib, in all CML phases.	Less sensitive mutations: V299L; F317L	
Bosutinib	BCR-ABL/Src	After intolerance or resistance to prior therapy.	T315I	(34–37^3,^^4^)
		For newly diagnosed-CML patients as first line TKI drug		
Ponatinib	BCR-ABL, Src, Akt	For all phase CML patients resistant or intolerant to prior TKI therapy.	Compound mutations in CML-AP (T315I/E453K) and CML-BP (T315I/F359C) patients	(38–42^5^)
		In presence of T315I BCR-ABL mutation in all CML phases		

^1^https://www.ema.europa.eu/en/documents/overview/glivec-epar-summary-public_en.pdf.

^2^https://www.accessdata.fda.gov/drugsatfda_docs/nda/2007/022068s000_LBL.pdf.

^3^https://www.accessdata.fda.gov/drugsatfda_docs/label/2017/20334s007s008lbl.pdf.

^4^https://www.ema.europa.eu/en/documents/overview/bosulif-epar-medicine-overview_en.pdf.

^5^https://www.accessdata.fda.gov/drugsatfda_docs/label/2016/203469s022lbl.pdf.

Ponatinib is a *third-generation* TKI employed for CML patients carrying the T315I mutation in BCR-ABL (BCR-ABL^T315I^) for which *first* and *second-generation* TKIs result inefficient (38). Unlike the other inhibitors mentioned above, Ponatinib inhibits the BCR-ABL^T315I^ activity due to its major affinity to modified amino acid I315 (52). In a recent clinical trial, its efficacy was demonstrated with the achievement of prolonged MMR and MCyR in patients intolerant to Dasatinib/Nilotinib or having T315I mutation (NCT01207440) (53). Adverse effects seen after Ponatinib-treatment are rash, abdominal pain, dryness of the skin, thrombocytopenia and increased lipase levels (39), thrombosis and other cardiovascular problems (54). For this reason, several studies evidence the need to use low doses of Ponatinib to treat CML patients resistant to other TKIs without impairing its efficacy (55, 56).

## 3 NEW BCR-ABL and Non-BCR-ABL TARGETED THERAPIES

Although TKIs have proven their clinical efficacy in the treatment of CML, the onset of resistance still represents a critical issue. In order to overcome the resistance, alternative therapies have been developed. For this reason, innovative BCR-ABL targeted or NON BCR-ABL targeted drugs can be considered as a valid alternative for CML patients resistant/intolerant to conventional treatment.

New approaches based on combination of drugs that act on different pathways can improve their effectiveness to obtain optimal response and reach TFR more quickly (57). We describe new specific drugs, focusing on the mechanisms of action, efficacy and safety. In detail, the molecules acting on BCR-ABL include Asciminib (ABL001), Olverembatinib (HQP1351), PF-114 and K0706 (58).

Non-BCR-ABL targeted therapies are employed to act on different pathways than those related to TKIs and/or promote LSCs eradication to overcome TKIs resistance in CML. Among these, we describe the action of inhibitors targeting farnesyl transferase (Lonafarnib and Tipifarnib), mammalian target of Rapamycin (mTOR, Rapamycin and Everolimus), Janus kinase 2 (JAK2), histone deacetylase (HDAC) and aurora kinase; peroxisome proliferator-activated receptor gamma (PPARγ) activators, hypomethylating agents and Omacetaxine. All drugs tested in clinical studies are summarized in **Table 2**.

**Table 2:** New BCR-ABL and NON BCR-ABL targeted therapeutic approaches for CML patients.

Drugs	Molecular target of the drugs	Indications or efficacy	Clinical Trials Number
**BCR-ABL targeted therapy**			
*Asciminib* (ABL001)	BCR-ABL1 Kinase	Resistance or failure to TKI Monotherapy/combination	NCT03595917 (Phase I)
		The combination therapy is promising in patients with T315I mutation	NCT03578367 (Phase II with *Imatinib)*
			NCT03595917 (Phase I with *Dasatinib*)
			NCT02081378 (Phase I with *Nilotinib*)
			NCT03106779 (Phase III with *Bosutinib*)
*Olverembatinib* (HQP1351)	BCR-ABL1	Resistance or failure to TKI	NCT03883087 (Phase II)
		Is efficacious in patients with T315I and other mutations	NCT03883100 (Phase II)
*Vodobatinib* (K0706)	BCR-ABL1	Resistance or failure to ≥ 3TKIs, except for patients carrying BCR-ABL T315I mutation	NCT02629692 (phase I/II)
*PF-114*	BCR-ABL1	Resistance or failure to ≥2GTKI	NCT02885766 (phase I/II)
		Is efficacious in patients with T315I mutations and other resistant mutations in BCR-ABL	
**NON BCR-ABL targeted therapy**			
*Tipifarnib* or *Zarnestra* (R115777)	Farnesyl transferase	Resistance or failure to TKI	NCT00040105 (phase I)
*Lonafarnib* (SCH66336)			NCT00047502 (phase I)
			NCT00038597 (phase II)
*Rapamycin*	mTOR	Resistance or failure to TKI	NCT00776373 (phase I/II)
*Everolimus*		In combination with DNA damaging agent etoposide	NCT00081874 (phase I/II)
			NCT00093639 (phase I)
*Ruxolitinib*	*JAK2/STAT5*	Resistance disease to eradicate the LSCs. In combination with Nilotinib in advanced or resistant disease	NCT01702064 (phase I)
			NCT02253277 (phase I)
		In combination with conventional TKIs and TFR	NCT03654768 (phase II)
			NCT03610971 (phase II)
*Panobinostat* (LBH589)	*Histone deacetylase*	Resistance or failure to TKI	NCT00449761(phase II)
			NCT00451035 (phase II)
*Tozasertib* (MK0457)	*Aurora kinase and BRC-ABL*	Resistance or failure to TKI	NCT00405054 (phase II)
		Is efficacious in patients with T315I	NCT00500006 (phase I)
*Danusertib* (PHA-739358)		Activity against BCR kinase including the gatekeeper T315I mutant	The European Clinical Trails
			Data Base (EudraCT number 2007-004070-18).
*Pioglityazone*	*PPARgamma*	Resistance disease to eradicate the LSCs.	NCT02888964 (phase II)
		In combination with Imatinib is promising and TFR	EudraCT 2009-011675-79
			NCT02889003 (phase II)
*Decitabine*	*DNA*	Resistance or failure to TKI	NCT01498445 Phase I/II
		Have effects both with monotherapy or combined with imatinib	NCT00042016 Phase II
			NCT00054431 Phase II
*Omacetaxine*	*Protein synthesis*	Resistance to ≥ 2 TKIs	NCT00462943 (phase II)
		Monotherapy in patients with BCR-ABL T315I mutation	NCT02078960 (Phase I/II)
			NCT00375219 (phase II)

### 3.1 BCR-ABL Targeted Therapy

#### 3.1.1 Asciminib

Asciminib is a potent, specific BCR-ABL kinase inhibitor that recognizes the myristoyl pocket contrary to conventional TKIs that target the ATP binding site (**Figure 1A**). Asciminib binds to the BCR-ABL oncoprotein, induces a conformational change in the myristoyl pocket leading to the inactive state of the kinase and its regulation (59). Its different mechanism of action could increase the efficacy especially in patients resistant or with therapeutic failure to conventional TKIs.

A combined approach of TKIs with Asciminib is important, having a different target therapy site.

In phase I studies (NCT03595917 and NCT02081378), Asciminib monotherapy showed the achievement of MCyR in 82% of chronic phase CML-patients who failed with three or more TKIs (57, 60), and at 12 months the incidence of MMR and CCyR were 48% and 70%, respectively (61). Clinical trials are ongoing to evaluate the efficacy of the combination of Asciminib with Imatinib (phase II study: NCT03578367), Dasatinib (phase I study: NCT03595917), Nilotinib (phase 1 study: NCT02081378) and Bosutinib (phase III study: NCT03106779). However, preliminary data confirm that this drug has greater efficacy compared to Bosutinib and high tolerability in CML-CP patients resistant or intolerant to ≥2 prior TKIs (62). Recently, the FDA approved Asciminib (Scemblix, Novartis AG) for patients in the chronic phase with failure to ≥2 TKIs or carrying T315I mutation (63 ^4^). In conclusion, the combination of Asciminib with ATP-competitive TKIs can be considered a valid therapeutic approach in CML patients with failure of more TKIs.

#### 3.1.2 Olverembatinib

Olverembatinib (HQP1351) is a new *third-generation* TKI, active against both wild type (wt) and mutated BCR-ABL (mut) isoforms. Recently, Jiang et al. demonstrate, in a phase I study in patients with CML resistant to current TKIs therapies, a long-lasting antitumor activity associated with high tolerability, particularly in subjects with T315I mutation (64). Preliminary data on patients with mean follow-up of 12.8 months showed that the treatment with HQP1351 promoted the achievement of a complete haematological response (CHR) in 94.5% of CML-CP and 84.6% of CML-AP patients versus baseline. In 69.1% of chronic phase subjects, the treatment induced MCyR compared to 42.9% in accelerated phase. A notable increase in CCyR was instead observed in 60.5% of patients with CML-CP compared to baseline, in the corresponding CML-AP patients was only 35.7%. These results were also confirmed in patients carrying BCR-ABL T315I mutation (64). Two Phase II clinical studies are ongoing in China in patients with CML-CP and CML-AP harboring T315I mutation to evaluate the efficacy of HQP1351 both on the MCyR and major hematological response, as well as its safety profile (NCT03883087 and NCT03883100). Non-severe clinical effects, such as thrombocytopenia, anaemia and leukopenia, were found in CML-CP and CML-AP patients (65).

#### 3.1.3 Vodobatinib and PF-114

*Vodobatinib (*K0706) and PF-114 are innovative ATP-competitive TKIs belonging to *third-* and *fourth-generation*, respectively. K0706 is a novel BCR-ABL TKI direct to wild-type and mutated isoforms although with less affinity for T315I mutation (66). A phase I/II study (NCT02629692) is ongoing to evaluate the efficacy and safety of K0706 in patients with comorbidities precluding the use of conventional *second-generation* TKIs or after ≥ 3 TKIs failure. The initial phase I results of this study showed disease progression in patients carrying BCR-ABL T315I mutation, therefore the enrolment of patients with this mutation was stopped (67).

PF-114 is a TKI similar to Ponatinib due to its chemical structure, capable of acting on both wt and mutated BCR-ABL including T315I. Unlike Ponatinib, PF-114 was designed not to inhibit the vascular endothelial growth factor receptor (VEGFR) in order to reduce cardiovascular effects in patients (68). 65 patients, resistant to *second-generation* TKIs or carrying the BCR-ABL^T315I^ mutation, were enrolled in a phase I/II clinical study to evaluate safety, pharmacokinetics and tolerability still ongoing (NCT02885766). In a phase I study, Turkina et al. achieved a complete hematologic response in 42.1% patients treated with PF-114 of which 37.5% were carriers of the T315I mutation. At different doses, mainly 200 and 300 mg, a complete MMR occurred in 11% of patients and a MCyR in 28.5% (69). However, the most effective dose was 300 mg with effective response in 41.6% of patients with BCR-ABL^T315I^. The most side effect of PF-114 is reversible grade 3 skin toxicity was found at 400 mg, in 11/12 patients (70).

In conclusion, PF-114 and K0706 have an acceptable safety profile in patients who are resistant and/or intolerant to ≥3 TKIs for K0706 and ≥ 2 for PF-114 (67, 70).

### 3.2 Non BCR-ABL Targeted Therapy

#### 3.2.1 Tipifarnib and Lonafarnib (Farnesyl Transferase Inhibitors)

Farnesyl transferase inhibitors (FTIs), as Tipifarnib or Zarnestra (R115777) and Lonafarnib (SCH66336), inhibit the activity of farnesyl transferase, acting on downstream targets such as the oncogene RAS and resulting in cell growth arrest (71, 72).

In a first pilot study conducted with Tipifarnib as monotherapy, a complete or partial haematological response was detected in 7/22 patients with a minor transient cytogenetic response in 4 of these (73).

Co-treatment with Tipifarnib, after previous Imatinib failure, induced a cytogenetic and overall haematological response in 36% and 76% of patients, respectively (NCT00040105). The combination was also well tolerated and had a selective activity for mutated kinase domains (74).

Regarding Lonafarnib, its efficacy was evaluated as monotherapy in a pilotal study on 13 patients with chronic and accelerated phase CML, finding a haematological response in only 15.3% (NCT00038597) (75). Subsequently, the combination with Imatinib was tested on a larger court of CML patients, finding an increase in its efficacy in 35% of patients, based on haematological and cytogenetic responses (NCT00047502) (76).

Using Lonafarnib as monotherapy only small benefits are seen in patients, while the combination can be used in patients unresponsive to TKI therapy (57).

#### 3.2.2 mTOR Inhibitors

The specific target of mTOR inhibitors is a serine/threonine kinase which is over-activated in CML. Rapamycin and Everolimus belong to this family. To date, only one clinical trial is currently ongoing for Rapamycin in combination with DNA-damaging agents (etoposide) (NCT00776373) (57). Everolimus blocks the constitutive activation of mTOR and makes cells more sensitive to Imatinib in combination therapy. Two clinical trials are underway to prove its safety and efficacy both as monotherapy and in co-treatment (NCT00081874 and NCT00093639).

#### 3.2.3 JAK2 Inhibitors

JAK2 inhibitors, such as Ruxolitinib, act on Janus kinase 2 (JAK2), an intracellular kinase commonly involved in phosphorylation and activation of STAT5 (77). There is considerable clinical interest in inhibiting JAK2 activity to deplete quiescent TKIs-resistant LSCs. The advantage of these inhibitors may be related to their ability to downregulate the JAK2/STAT5 pathway which plays a crucial role in the proliferation and survival of CML LSCs (78). A promising therapeutic approach given by the combination of Ruxolitinib and Nilotinib has been evaluated in adult CML-CP patients in a phase 1 study. Preliminary data confirm that the combination Ruxolitinib and Nilotinib is safe and well tolerated with encouraging molecular responses (NCT01702064; NCT02253277). However, these data need to be validated in a phase II study (79). An ongoing phase II study has the primary objective to evaluate the molecular responses after the combination of Ruxolitinib plus conventional TKIs (NCT03654768). The effectiveness on TFR of adding Ruxolitinib to TKIs is currently under a phase II study (NCT03610971).

In conclusion, the main advantage in the use of this therapeutic approach with JAK2 inhibitors may be associated with the eradication of LSCs responsible for BCR-ABL independent resistance.

#### 3.2.4 Histone Deacetylase Inhibitors

Emerging alternative therapies are Histone deacetylase inhibitors (HDACis), involved in the epigenetic modifications that regulate the acetylation state of histones. Zhang et al. proved in CML cells that the co-treatment of HDACi with Imatinib downregulated the expression levels of genes involved in the maintenance of CML LSCs, such as Wnt/β-catenin (80). Panobinostat is a HDACi, whose efficacy was demonstrated both *in vitro* including on cells carrying BCR-ABL^T315I^ and *in vivo* as reported in two phase 2 clinical trials (NCT00449761 and NCT00451035) (57). In patients CML-CP resistant to ≥ 2 TKIs, Panobinostat monotherapy didn’t induce MCyR but only in 3% a complete haematological response was observed (81). Finally, emerging evidence has shown that HDACis may have promising action as a combination therapy in patients with TKIs failure compared to the reduced efficacy in monotherapy (82).

#### 3.2.5 Aurora Kinase Inhibitors (AURCis)

Aurora kinase inhibitors act on the serine-threonine kinases of Aurora family proteins which are key regulators of cell division whose dysregulation results in DNA alterations and cellular transformation. Tozasertib and Danusertib are Aurora kinase inhibitors. The first drug is known to avoid CML progression and even lead to a change from advanced to chronic phase in CML patients, including those carrying BCR-ABL^T315I^. The second agent is characterized by a dual role, since it can inhibits both Aurora kinase family and BCR-ABL, including T315I (82). As monotherapy, a phase II study was performed in CML-CP patients with T315I mutation (NCT00405054). Overall, a major cytogenetic response was achieved in 8% of patients, unconfirmed complete or partial response in 6% and only 13.3% had a complete haematological response (83). While, the combination with Dasatinib was used in phase I study to determine safety and efficacy of Tozasertib (NCT00500006).

Danusertib is a drug with an acceptable safety profile, as demonstrated in a clinical trial (EudraCT number 2007-004070-18). When it was administered as a single drug, complete molecular and cytogenetic responses appeared to be constant over time in all patients carrying T315I BCR-ABL mutation (84).

#### 3.2.6 PPARgamma (PPARγ) Activators

Glitazones are *PPARgamma* (PPARγ) activators utilized for diabetic patients. However, they can be successfully employed in CML therapy, especially to counteract the persistence of CML LSCs. PPARγ reduce the expression of STAT5 and its downstream targets which are involved in the survival of quiescent CML LSCs. As shown in clinical trials (NCT02888964; EudraCT 2009‐011675‐79), the combination of Pioglitazone with Imatinib was well tolerated in CML patients with a better molecular response (MR 4.5) in 56% versus 23% found in patients treated with Imatinib alone (85). Finally, there is a current phase II study that aims to further characterize the use of Pioglitazone in patients with TKIs failure after first TFR to proceed with the second discontinuation attempt (NCT02889003). In summary, this combination constitutes a potential strategy for the eradication of quiescent LSCs.

#### 3.2.7 Hypomethylating Agents

High methylation levels in ABL play a critical role in the progression from CML-CP to CML-BP (86). Since DNA methylation correlates with the progression, the use of hypomethylating agents constitutes an alternative therapy in combination with TKIs in CML. Among these drugs, Decitabine in combination with Dasatinib induced complete haematological response in 48% of patients, achieving MCyR and MMR in 44% and 33% of patients, respectively (NCT01498445) (87). Severe myelosuppression was an adverse effect occurring in patients treated with this methylating agent (88). Two clinical trials of phase II are evaluating the efficacy of Decitabine both as monotherapy and in combination with Imatinib in patients who had no benefit from conventional therapy and disease progression (NCT00042016; NCT00054431). Venetoclax, a selective BCL2 inhibitor, is approved in combination with hypomethylating agents for newly diagnosed Acute Myeloid Leukemia (AML) (89). Preclinical studies demonstrated a synergism of Venetoclax in combination with BCR-ABL TKI in the eradication of LSCs in CML-BP (90). Indeed, the overall response rate (ORR) was 75% in CML-BP and 43% in AML patients (91).

#### 3.2.8 Omacetaxine

A new drug relevant for the treatment of chronic or accelerated phase CML is Omacetaxine, considered a *third generation* TKI capable of inhibiting protein synthesis (**Figure 1B**). The efficacy of Omacetaxine has been evaluated in several clinical trials in patients resistant to more than 2 TKIs (NCT02078960, phase I/II and NCT00462943, phase II) and carriers of the T315I mutation (NCT00375219, phase II). FDA guidelines indicate this drug effective for patients intolerant/resistant to ≥2 TKI, including those with BCR-ABL^T315I^ (68). Based on the results obtained by Cortes *et al.*, 18% of patients with CP-CML achieved persistent MCyR for 12.5 months; while only 14% of CML-AP patients had a major haematological response for 4.7 months (92). Therefore, long-term administration of Omacetaxine results in prolonged benefits. On the other hand, the main adverse effect is the haematological toxicity induced in 10% of CML-CP patients and in 5% of CML-AP patients resulting in discontinuous treatment (92).

## 4 Innovative Potential Therapeutic Approaches in CML Therapy

### 4.1 Antioxidants and Their Role in CML Therapy

The activity of BCR-ABL oncoprotein is linked to ROS production leading to the onset of new mutations (93). This “self-mutagenesis” favors the progression of CML from CP to BP (94). Usually, alterated levels of antioxidant enzyme superoxide dismutase (SOD) (95), thiobarbituric acid reactive substances (TBARS), total lipid hydroperoxides (96) and higher plasmatic malondialdehyde levels are observed in CML patients compared to healthy subjects (97). Antioxidants could be a useful weapon to reduce oxidative stress and to hinder the onset of new BCR-ABL mutations, as demonstrated *in vivo* with xenograft mice (98). Ghalaut *et al.* detected a reduction in nitrosative stress (NO) levels following the addition of turmeric to the diet of patients treated with Imatinib (99) proposing the antioxidants as possible new therapeutic approach. The anti-proliferative and pro-apoptotic properties of curcumin are known, as demonstrated in K562 leukemia cells (100). It was also proved that curcumin was able to increase PTEN and inhibit BCR-ABL activity *in vitro*, while the tumor size was reduced in xenograft model (101). Wu *et al.* proposed two curcumin derivates, C086 and C817, as a potential therapeutic approach to counteract Imatinib-resistance (102, 103). Curcumin derivates are capable of promoting apoptosis in cells carrying the following mutations: Y253F, Q252H and, including, T315I. Moreover, a reduced survival of CML progenitor/stem cells, obtained from patients ‘bone marrow, was observed after the treatment with the same derivatives of curcumin (102, 103). These agents may constitute a new potential therapeutic approach to overcome the persistence of LSCs resistant to TKIs.

Although several antioxidants play an essential role in reducing oxidative damage, some of them at high concentrations show pro-oxidant action and induce death in cancer cells (104) such as resveratrol (RES) which has a dual effect in a dose-dependent manner (105). The latter is a compound that at low doses (20 µM) it is able to reduce oxidative stress (106) while at higher doses (60μmol/L) it induces apoptosis in K562 cells depending on the duration of the treatment (107).

Damiano et al. evaluated the antiproliferative effects of combining TKIs (Nilotinib or Dasatinib) with different antioxidants including RES, δ-tocotrienol (δ-TOCO) and a new recombinant mitochondrial manganese containing superoxide dismutase (rMnSOD) in K562 cells. The results suggested that the combination increased the action of inhibitors and induced the cytotoxic effect through the production of ROS confirming the potential use of antioxidants in CML (108).

### 4.2 Oncolytic Virus

The development of a specific target-therapy is a major focus of cancer research. In recent years, oncolytic virus (OV) therapy or virotherapy has become a promising alternative strategy through the use of wild type or genetically modified viruses (109).

In detail, the most exploited oncolytic viruses in virotherapy are: adenovirus, herpesvirus, herpes simplex (HSV), measles virus, parvovirus and reovirus (110–113). For human adenovirus and herpes simplex 1 (HSV-1), additional genetic modifications are required in order to both mitigate virulence and improve specificity and safety (114, 115). Non-human viruses don’t need to be modified due to their species-specific infection capacity (116–118).

In our previous studies, we demonstrated the efficacy of the Caprine herpesvirus type 1 (CpHV-1) in promoting the death of different tumor cell lines. However, it was not able to reduce the viability of K562 probably due to a lower permissiveness of these cells to this type of caprine virus (116).

To improve the efficacy of virotherapy, viruses are often engineered or modified with tumor suppressor or pro-apoptotic genes (119). As suggested by *in vitro* and *in vivo* studies, adenoviruses with chimeric Ad5/11 fiber expressing Beclin-1 could be a promising approach in CML therapy (120).

By overexpressing Beclin-1 protein in chimeric adenovirus 5/11 (SG511-BECN), Tong *et al.* induced autophagy in primary cells obtained from Imatinib-resistant CML patients.

Increased survival has also been shown in xenografted mice after treatment with SG511-BECN (121).

Li et al. used oncolytic viruses in combination with other anticancer drugs to demonstrate their efficacy in CML. The data confirmed a high antitumor action in multidrug- resistant CML cells after combined treatment with SG511-BECN and chemotherapy agents (122). In conclusion, these promising results reinforce the idea of employing this new type of therapeutic approach also for CML disease.

### 4.3 Exosomes as Biomarkers and Drug Carriers in CML

Exosomes are membrane-bound extracellular vesicles (EVs) that carry both RNA and other bio-molecules, such as lipids and proteins; they are often found in biological fluids, including blood, urine and cerebrospinal fluid (123).

The presence of BCR-ABL is now also known in exosomes obtained from CML cells (124), therefore these EVs could be useful for the identification of new biomarkers for CML. One of these is miR-140-3p, upregulated in CML patients and involved in the inflammatory process (125).

Exosomes can also be used as drug carriers in fact Bellavia et al. employed exosomes expressing Interleukin 3 (IL-3), whose receptor is usually overexpressed in CML blasts, to deliver Imatinib or BCR-ABL siRNA. These exosomes thus loaded were capable of inhibiting cancer cell growth both *in vitro* and *in vivo* (126). While these data are promising, further studies are needed to demonstrate and test the efficacy of exosomes as a vehicle for CML-specific drugs and siRNA.

### 4.4 Active Compounds From Marine Organisms

Marine organisms are able to produce metabolites with bioactivities useful for the treatment or prevention of human pathologies (127–130). There are currently on the market fourteen compounds derived from marine species used for various types of cancer (such as multiple myeloma, leukemia, lymphoma, breast, ovarian, lung and urothelial cancer), hypertriglyceridemia, pain and infections (https://www.midwestern.edu/departments/marinepharmacology/clinical-pipeline.xml). In addition to those on the market, other marine compounds/extracts showed antiproliferative activities against various cancer cells (131–133). Anticancer is the most frequent activity identified for marine derived compounds, maybe because these molecules have defensive roles in the natural environments. In addition, there is great interest in marine natural products because of their novel chemical structures that often have no other equivalent in terrestrial habitats (~70% of their structural scaffolds are only found in marine organisms).

Most of the marine compounds active against chronic myelogenous leukemia cells derives from sponges. The National Cancer Institute screened about 90,000 extracts of terrestrial and marine plants, and invertebrates on its prescreen platform of 60 human cell lines including the K562 and the HL-60 cell lines (134). Data showed that different phyla, including Annelida, Bryozoa, Chlorophyta, Chordata, Cnidaria, Cyanophyta, Echinodermata, Mollusca, Phaeophyta, Porifera, Rhodophyta and Tracheophyta (Mangrove) and 620/9945 extracts had antileukemia properties. Among the screened organisms, Porifera showed selectivity against leukemia cell lines (134). Regarding the purified compounds from sponges with activity against CML, there are gombamide A, compound 1-8, Lembehyne B, Heteronemin, Smenospongine, Aaptamine, chujamides A and B (135–142).

In particular, gombamide A is a peptide obtained from *Clathria gombawuiensis*, a native sponge of Korean waters (135). It was proved that gombamide A had cytotoxicity effects against K562 cells with LC50 of 6.9 µM (135). Compound 1-8, extracted from the sponge *Coscinoderma* sp. of Chuuk Island (Micronesia) (136), showed to be cytotoxic against K562 leukemia cells with LC50 value of 0.9-5.5 µM. Lembehyne B, an acetylenic alcohol obtained from the sponge *Haliclona* sp. living in the Indonesian waters (137), exhibited cytotoxic properties against K562 cells showing an IC50 of 3 µM (138). In addition, Lembehyne B induced apoptosis and phosphatidylserine externalization on the plasmatic membrane after treatment. Heteronemin is a sesterterpene derivative isolated from the sponge *Hippospongia* sp (139). which showed strong anticancer activity against K562 cells with EC50 value of 0.41 ± 0.08 mg/mL. Smenospongine is a compound extracted from the marine sponge *Dactylospongia elegans* which was active against K562 cells and promoted cell cycle arrest when tested at 5-15 µM (140). In addition, Smenospongine was able to block the progression of cell cycle by increasing p21 levels and reducing Rb phosphorilation in K562 cells. Aaptamine is a marine-derived alkaloid isolated from the sponge *Aaptos suberitoids* (141) and able to inhibit CML K562 cell proliferation with a GI50 of 10 μM. Aaptamine also induced the arrest of cell cycle at G2/M phase and a higher p21 levels, as demonstrated by protein analyses in K562 cells. Finally, the isolation from the sponge *Suberites waedoensis* of chujamides A and B was reported by Song and co-authors (142). Chujamides A and B are cyclic cysteine bridged peptides, with cytotoxicity toward K562 cells with LC50 values of 37 µM and 55.6 µM, respectively.

Other promising activities derive from marine microalgae, more manageable to grow in small or large photobioreactors compared to sponges and representing an eco-friendly and eco-sustainable source of new compounds from marine environments still poorly explored (130, 133, 143–145). Carbenolide was isolated from the dinoflagellate *Amphidinium* sp. GA3P and was able to induce apoptosis in K562 cells by inhibiting DNA topoisomerase I and II showing an IC50 of 30 ng/mL (146). Additionally, it was shown that Carbenolide raised lifespan in mice implanted with P-388 leukemia cells (147). Recently, Atasever-Arslan and collaborators (148) tested a serious of microalgae for possible cytotoxicity against both HL60 and K562 cell lines. *Stichococcus bacillaris*, *Phaeodactylum tricornutum*, *Microcystis aeruginosa* and *Nannochloropsis oculata* extracts were found active against one or both cell lines when screened at 1-500 µg/mL. In particular, *S. bacillaris*, *M. aeruginosa* and *N. oculata* extracts were able to induce apoptosis in K562 cells as demonstrated by Annexin V, PI and DNA fragmentation analyses. *S. bacillaris* also increased phospho-p38 MAPK protein levels in K562 cells. Atasever-Arslan et al. (148) chemically characterized the composition of oils of these microalgal species by gas chromatography-mass spectrometry (GC-MS) and found 206 compounds. In the active extracts, twelve of these compounds were identified. Hence, these twelve molecules were further analyzed for docking analysis against various key intracellular proteins and results showed that five of them had *in silico* possible antileukemic activities (e.g. inhibitors of key proteins).

## 5 Conclusion

Targeted therapy using TKIs is considered the conventional treatment in CML patients. Some limitations of TKIs use are the high healthcare costs of the drugs, the continuous molecular monitoring of patients for the identification of new mutations and the persistence of LSCs that induce resistance to TKIs. At present, the development of new therapeutic approaches aims at increasing survival, improving quality of life and achieving successful TFR after TKIs discontinuation. BCR-ABL targeted and non BCR-ABL targeted therapies, alone or in combination with conventional inhibitors, constitute a new valid approach to achieve better molecular, haematological or cytogenetic responses and to attempt TFR. Preliminary data *in vitro* and *in vivo* results based on the use of oncolytic viruses and engineered exosomes as drugs carrier prove that they could be employed to selectively eradicate CML cells. The ability of antioxidants in combination with TKIs could be a future approach to be employed for CML therapy. Although further investigation is required to better understand the molecular mechanism of these new agents, they represent promising options that could pave the way for new therapeutic approaches for CML patients.

## Author Contributions

Conceptualization, EA, SD, CLo, CLa and RC; writing original draft preparation, EA and CC; supervision, AG, SM, MQ, CC and RC; project administration, RC and FP; funding acquisition, CC., SM and RC. All authors contributed to the article and approved the submitted version.

## Funding

This research was supported by: The Italian Ministry of Health progetto di Ricerca Corrente (M4/7) “Identificazione di nuovi approcci per la diagnosi e terapia del mesotelioma pleurico” and grant from Italian Association Leukemia and Lymphoma (A.I.L.) - Caserta - ONLUS “Valentina Picazio”. Human Health Foundation Onlus (http://www.hhfonlus.org).

## Conflict of Interest

The authors declare that the research was conducted in the absence of any commercial or financial relationships that could be construed as a potential conflict of interest.

## Publisher’s Note

All claims expressed in this article are solely those of the authors and do not necessarily represent those of their affiliated organizations, or those of the publisher, the editors and the reviewers. Any product that may be evaluated in this article, or claim that may be made by its manufacturer, is not guaranteed or endorsed by the publisher.

## Footnotes

1
https://www.ema.europa.eu/en/medicines/human/EPAR/tasigna#authorisation-details-section.

3
https://www.accessdata.fda.gov/drugsatfda_docs/nda/2006/021986_022072_SprycelTOC.cfm.

4 https://www.fda.gov/drugs/resources-information-approved-drugs/fda-approves-asciminib-philadelphia-chromosome-positive-chronic-myeloid-leukemia.
